# CircNRIP1 Exerts Oncogenic Functions in Papillary Thyroid Carcinoma by Sponging miR-653-5p and Regulating PBX3 Expression

**DOI:** 10.1155/2022/2081501

**Published:** 2022-05-19

**Authors:** Lijun Fu, Jia Huo, Habibullah Fitrat, Yujing Kong, Lingyu Zhang, Chaoyang Shang, Guoquan Li, Feihong Ji, Xinghao Fu, Xinguang Qiu

**Affiliations:** ^1^Department of Thyroid Surgery, The First Affiliated Hospital of Zhengzhou University, No. l Construction of East Road, Erqi District, Zhengzhou, 450052 Henan, China; ^2^Department of Radiotherapy, The First Affiliated Hospital of Zhengzhou University, No. l Construction of East Road, Erqi District, Zhengzhou, 450052 Henan, China

## Abstract

**Background:**

Circular RNA circ_0004771 (termed circNRIP1) was identified by RNA-Seq previously and was elevated in papillary thyroid carcinoma (PTC) tissues. A series of studies also showed that circNRIP1 was upregulated in some tumors and could promote the malignant progression of tumors. This research intended to focus on the role of circNRIP1 in PTC progression and explore the mechanisms underlying circNRIP1 functions.

**Methods:**

RT-PCR or western blot determined circNRIP1, miR-653-5p, and pre-B-cell leukemia homeobox 3 (PBX3) expression. EdU, CCK-8, Tunel, and transwell assays determined cell proliferation, apoptosis, invasion, and migration, respectively. Luciferase reporter assay, RNA immunoprecipitation (RIP), and RNA pull down assays clarified the target relation between miR-653-5p and circNRIP1 or PBX3. Xenograft models were applied to explore the role of circNRIP1 *in vivo*.

**Results:**

circNRIP1 significantly increased in PTC tissues and PTC cell lines than that in normal ones. Higher circNRIP1 expression was associated with the TNM stage and poorer overall survival. circNRIP1 knockdown attenuated the malignant progression of PTC, specifically by inhibiting proliferation and invasion/migration and promoting apoptosis. circNRIP1 was a miR-653-5p sponge; miR-653-5p knockdown reversed the suppressive role of circNRIP1 silence in PTC progression. PBX3, a target of miR-653-5p, was positively medicated through circNRIP1 via competitively sponging miR-653-5p. Knockdown of circNRIP1 attenuated the PTC tumor progression via miR-653-5p/PBX3 axis.

**Conclusion:**

Silencing of circNRIP1 suppressed PTC development via miR-653-5p elevation and PBX3 reduction, providing a novel perspective for understanding PTC pathogenesis.

## 1. Introduction

Thyroid cancer occupies approximately 1% of all malignant tumors in the whole body, mainly including papillary carcinoma, follicular carcinoma, undifferentiated carcinoma, and medullary carcinoma [1]. Papillary thyroid carcinoma (PTC) with lower malignancy and better prognosis is the most common of all [2]. PTC can occur at any age, especially in young adults [3]. Although the specific pathogenesis of PTC is still unclear, countless evidences show that gene factors exert vital influences on the PTC pathogenesis [4, 5]. Therefore, the molecular mechanism of PTC occurrence and development and its influence on tumor progression has always been a hot topic.

Recently, with advancement of RNA sequencing (RNA-Seq) technique, novel endogenous circular noncoding RNAs (circRNAs) have drawn much attention in the field of gene therapy [6]. circRNAs are covalently linked by the 5′ and 3′ ends of linear RNA precursor and have the characteristics of closed-loop structure [7]. Compared with miRNAs and lncRNAs, circRNAs have the advantages of high tissue expression specificity, high conservation, and stable structure, which reflect that circRNAs can have more superior functions than miRNA and lncRNAs [8]. Multiple circRNAs regulate cell proliferation, apoptosis, differentiation, and development in different types of cells [9, 10]. In tumor system diseases, circRNAs are involved in the occurrence, development, metastasis, and prognosis of different tumors, including lung cancer, gastric cancer, colon cancer, and many other tumor diseases [11, 12]. Some circRNAs exert regulatory roles in PTC; for instance, circRUNX1 could enhance progression and metastasis of PTC via regulating cell proliferation and invasion [13]. Therefore, targeting circRNAs may be an important strategy to mediate the occurrence, progression, and tumor size of PTC, since they exert crucial functions in PTC cell proliferation, apoptosis, and differentiation.

Identifying the key genes in PTC occurrence and development is critical for the diagnosis and treatment of PTC. Previously, quite a lot of dysregulated circRNAs were discovered in PTC tissues, serving as vital roles in PTC progression [14, 15]. We screened some circRNAs that were differentially expressed in PTC from previous RNA-Seq analysis [16]. Through experiments and literature review, hsa_circ_0004771 (also termed as circNRIP1), which was identified as highly expressed in PTC tissues, attracted our attention. The oncogenetic role of circNRIP1 has been identified in gastric cancer, osteosarcoma, colorectal cancer, nasopharyngeal carcinoma, ovarian cancer, cervical cancer, and esophageal squamous cell carcinoma [17–23]. In PTC, a previous report by Li et al. revealed upregulated circNRIP1 in PTC [24]. Nevertheless, the specific role and functions of circNRIP1 in PTC are still elusive.

Herein, we first determined circNRIP1 level in 68 cases of PTC tissues and adjacent tissues. Next, through circNRIP1 expression intervention in different PTC cells, we clarified the role of circNRIP1 in PTC cell proliferation, apoptosis, invasion/migration, and tumor size *in vitro* and *in vivo*. Finally, we verified the molecular mechanism of tumor promoting effect of circNRIP1 in PTC.

## 2. Methods

### 2.1. Patients

A total of 68 PTC and adjacent normal tissues were obtained from PTC patients who underwent surgery in the First Affiliated Hospital of Zhengzhou University from May 2018 to July 2020. The clinical characteristics of these patients are shown in Table 1. This study was authorized by the Ethics Committee of the First Affiliated Hospital of Zhengzhou University, and all the patients signed the informed consents voluntarily.

### 2.2. Cell Culture and Transfection

The Nthy-ori-1 and PTC cell lines (IHH-4, SW579, TPC-1, BCPAP, and K-1) were purchased from Shanghai Institute of Cell Biology and cultured in RPMI-1640 medium (Sigma, USA) containing 10% fetal bovine serum (FBS, Sigma, USA) and 1% antibiotics (Sigma, USA). TPC-1 and K-1 cells were transfected with si-Ctrl, si-circ, NC-inhibitor, miR-653-5p inhibitor, sh-Ctrl, sh-PBX3, pcDNA3.1, and pcDNA3.1/PBX3 plasmids utilizing Lipofectamine® 2000 (Thermo Fisher Scientific, USA) under the manufacturer's instructions.

### 2.3. qRT-PCR Analysis

Total RNA was extracted from PTC tissues and cells with Trizol (Invitrogen, USA). SYBR Green RT-PCR SuperMix UDG reagents (Invitrogen, USA) and the MX3000P system (Stratagene, USA) were adopted to amplify cDNA. 18S was as the control of circNRIP1, while U6 was as the internal control of miR-653-5p. Specific primers are shown in Table 2.

### 2.4. CCK-8 Assay

The cells in each group were made into cell suspension using DMEM with 10% FBS and inoculated into a 96-well plate at a density of 2 × 10^3^ cells per well. After 24, 48, 72, and 96 h of incubation, 10 *μ*l CCK-8 solution (Dojindo, Japan) was added into each well. Then the cells were incubated in the dark for another 2 h. The absorbance value at 450 nm was measured and recorded.

### 2.5. EdU Incorporation Assay

EdU incorporation assay was used to detect cell proliferation. In brief, after 48 h of incubation, add 100 *μ*l medium with 50 *μ*M EdU to the TPC-1 and K-1 cells for another 2 h of incubation at 37°C. Then, the cells were fixed with 4% paraformaldehyde. The nuclei were stained with DAPI for 30 min. Finally, observe the EdU-positive cells under the microscope (Olympus) for calculating the percent.

### 2.6. Tunel

Apoptosis in tumor cells was detected by TdT-mediated dUTP Nick-End Labeling (Tunel) via an In situ Cell Death Detection kit (Roche, Germany). The kit was used according to the manufacturer's protocol.

### 2.7. Western Blot Analysis

Proteins were extracted using RIPA buffer (Invitrogen) from cells and tissues. The concentrations were determined with BCA kit. The primary antibodies against Bax (ab32503, 1 : 1000), Bcl-2 (ab32124, 1 : 1000), PBX3 (ab109173, 1 : 1000), and GAPDH (ab9485, 1 : 2500) and the secondary antibody (ab96899, 1 : 1000) were obtained from Abcam (Cambridge, CA, USA). Chemiluminescence detection system was used to detect the protein content.

### 2.8. Transwell Assay

Transwell chamber precoated with or without Matrigel was applied to measure cell migration and invasion. Briefly, TPC-1 and K-1 cells were placed in the superior chamber, and DMEM medium containing 10% FBS was placed in the lower chamber. After 24 h (migration) or 48 h (invasion) of incubation, the cells were fixed and furtherly dyed with crystal violet. The cell counting was performed with the microscope.

### 2.9. Subcellular Fractionation

RNA in the cytoplasm and nucleus were extracted from TPC-1 and K-1 cells with Paris Kit (ambion, USA). The relative expression of circNRIP1 was determined by qRT-PCR. GAPDH and U1 were as the control for cytoplasm and nucleus, respectively.

### 2.10. Luciferase Reporter Assay

circNRIP1 WT/Mut and PBX3 WT/Mut were designed upon the complementary sites of miR-653-5p in circNRIP1 or PBX3 3′-UTR sequence and cotransfected with TPC-1, K-1, and HEK-293T cells, respectively. After 24 h, the luciferase activity was determined using luciferase reporter system (Promega, USA).

### 2.11. RNA Pull down Assay

The Biotin circNRIP1 WT, Biotin circNRIP1 Mut, or Biotin Ctrl was overnight incubated with streptavidin beads (Thermo Fisher Scientific), respectively. The TPC-1 and K-1 cell lysates were transfected with the bead-biotin complex for 2 h. Then, the RNA was extracted, and the miR-653-5p relative enrichment was determined using qRT-PCR.

### 2.12. RIP Assay

RIP assay was carried out with Magna RIP kit (Millipore). The TPC-1 and K-1 cell lysates were incubated with Ago2 and IgG antibodies (Millpore). Then, the RNA was extracted and the miR-653-5p, circNRIP1, and PBX3 relative enrichments were determined using qRT-PCR.

### 2.13. Mouse Xenograft Assay

Ten male nude mice were obtained from Shanghai Animal Laboratory Center (Shanghai, China) and divided into the si-Ctrl group (*n* = 5) and the si-circ-1# group (*n* = 5). TPC-1 cells transfected with si-Ctrl or si-circ-1# were injected subcutaneously into the hip back of mice in the si-Ctrl group and the si-circ-1# group, respectively. The volume of the tumor was determined every week. Five weeks later, the animals were sacrificed for the tumors and the weight was recorded. In addition, the expression levels of circNRIP1, miR-653-5p, and PBX3 protein were determined in tumor tissues. The experiment conformed to the guide of using and caring of animals and was approved by the Ethics Committee of the First Affiliated Hospital of Zhengzhou University.

### 2.14. Statistical Analysis

SPSS 18.0 was adopted to carry out the statistical analysis. Measurement data were shown as the mean ± SD and analyzed by Student's *t*-test between two groups or one-way ANOVA among three groups. Count data Kaplan-Meier plot and log-rank test were performed to analyze the overall survival of patients. Enumeration data were expressed by percentage (%) and analyzed by *χ*^2^ test. *p* < 0.05 was considered significant.

## 3. Results

### 3.1. circNRIP1 Is Induced in PTC and circNRIP1 Knockdown Attenuates the Malignant Progression of PTC

**Figure 1:**
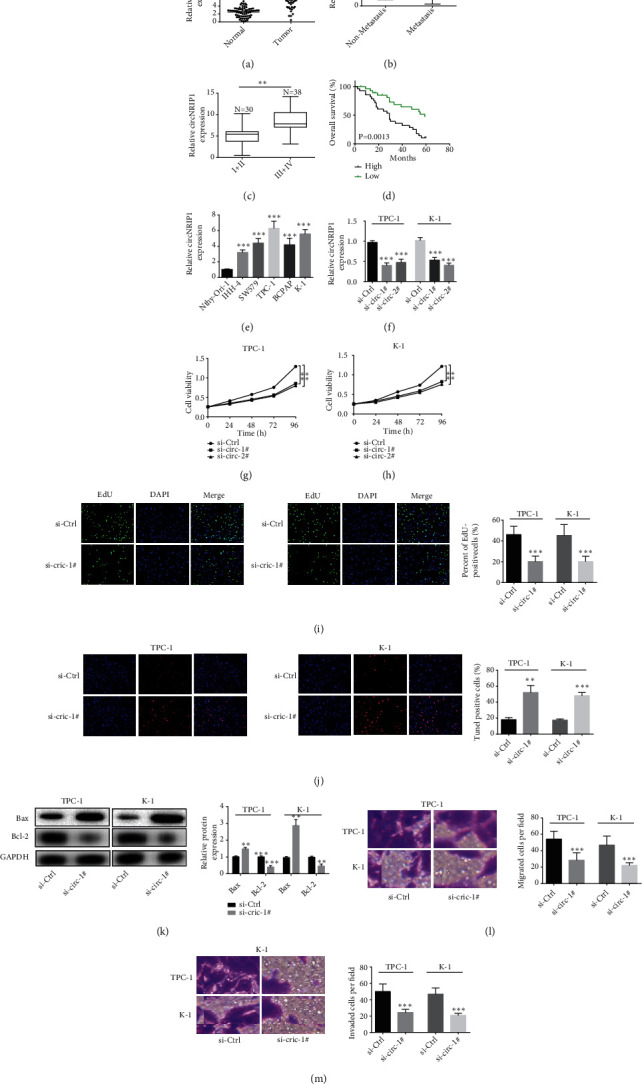
circNRIP1 is increased in PTC and circNRIP1 knockdown attenuates the malignant progression of PTC. (a) circNRIP1 expression in PTC tissues was determined by qRT-PCR assay. (b) circNRIP1 expression in PTC tissues with or without distant metastasis was detected using qRT-PCR assay. (c) circNRIP1 expression in PTC tissues with different TNM stages was evaluated with qRT-PCR assay. (d) The relationship between circNRIP1 expression and overall survival was estimated using Kaplan-Meier analysis. (e) circNRIP1 expression in PTC cell lines was determined by qRT-PCR assay. (f) The success of knockdown of circNRIP1 with si-circ-#1/2 in PTC cells was confirmed by qRT-PCR analysis. (g–i) CCK-8 and EdU incorporation assay were used to detect the cell proliferation. (j) Tunel staining was used to evaluate the apoptosis of tumor cells. (k) Western blot was conducted to evaluate the apoptosis biomarkers. (l, m) The cell migration and invasion abilities were detected with transwell assay. ^∗∗^*p* < 0.01;  ^∗∗∗^*p* < 0.001.

To investigate the circNRIP1 expression in human PTC specimens, we first collected PTC tissues and controls and identified circNRIP1 level using qRT-PCR assay. circNRIP1 was elevated in PTC tissues (Figure 1(a)). Besides, circNRIP1 in PTC with distant metastasis showed higher level than that without distant metastasis (Figure 1(b)). In addition, circNRIP1 expression seemed to have a closed correlation with TNM stage, as patients in the advanced stage usually showed higher circNRIP1 expression levels than those in the early stage (Figure 1(c)). Furthermore, higher circNRIP1 expression was associated with poorer overall survival (Figure 1(d)). Consistently, we also discovered that circNRIP1 expression increased in PTC cell lines (Figure 1(e)). As they exhibited higher circNRIP1 expression, TPC-1 and K-1 cell lines were then used for the following assays. Subsequently, si-circ-#1 and si-circ-#2 were transfected into TPC-1 and K-1 cells for circNRIP1 inhibition. The success of knockdown in PTC cells was confirmed by qRT-PCR analysis (Figure 1(f)). Subsequently, we conducted CCK-8 and EdU incorporation to measure cell proliferation. The results demonstrated that circNRIP1 knockdown attenuated cell viability (Figures 1(g)–1(i)). Besides, Tunel staining illustrated the increase in the proportion of Tunel-positive cells under circNRIP1 knockdown (Figure 1(j)). Also, western blot was conducted to evaluate the apoptosis biomarkers, and the results showed Bax was induced while Bcl-2 was decreased after silencing circNRIP1 (Figure 1(k)). In addition, knockdown of circNRIP1 decreased the migrated and invaded cells as exhibited by transwell assay (Figures 1(l) and 1(m)).

### 3.2. circNRIP1 Is Directly Bound to miR-653-5p

**Figure 2:**
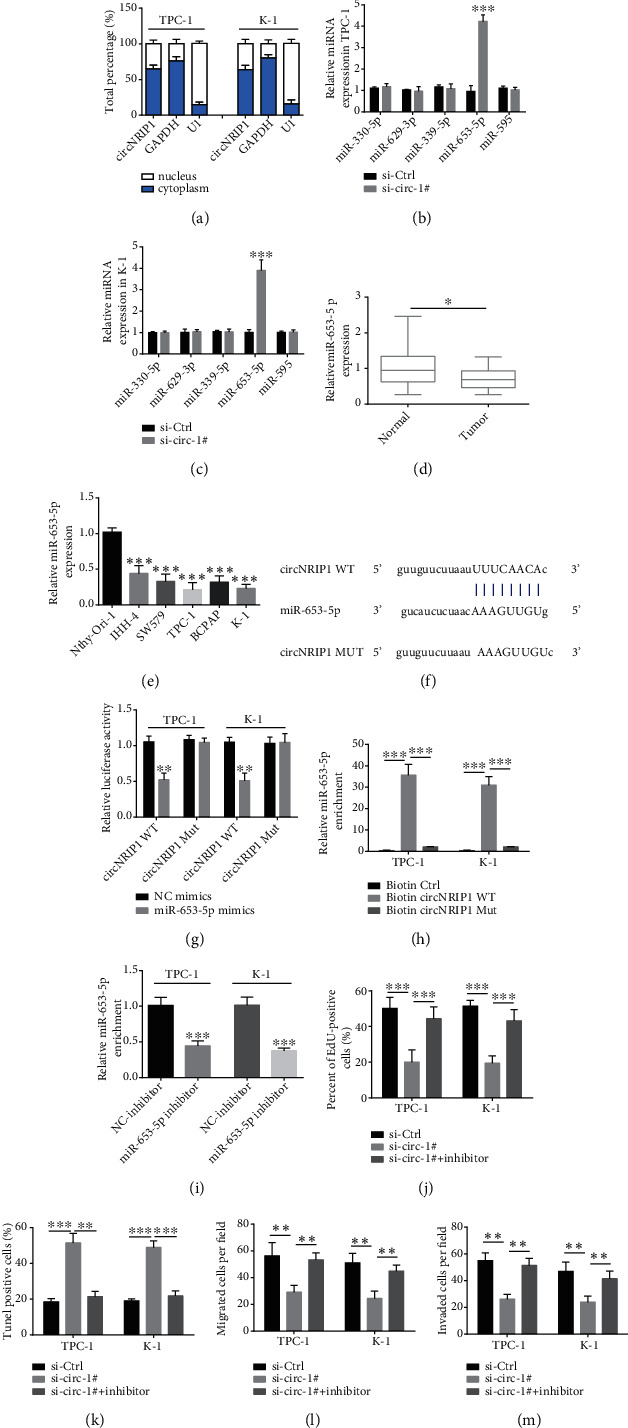
circNRIP1 directly binds to miR-653-5p. (a) The location of circNRIP1 was verified by the subcellular fractionation assay. (b, c) The potential binding microRNA expressions after knockdown of circNRIP1 were determined by qRT-PCR analysis. (d, e) The miR-653-5p expressions in PTC tissues and cell lines were determined by qRT-PCR assay. (f) The binding sites of miR-653-5p in circNRIP1. (g, h) Luciferase reporter assay and RNA pull down assay were carried out to investigate the physical connection between circNRIP1 and miR-653-5p. (i) The success of miR-653-5p inhibition with miR-653-5p inhibitor in PTC cells was confirmed by qRT-PCR analysis. (j) EdU incorporation assay was used to detect the cell proliferation. (k) Tunel staining was used to evaluate the apoptosis of tumor cells. (l, m) The cell migration and invasion abilities were detected with transwell assay. ^∗^*p* < 0.05;  ^∗∗^*p* < 0.01;  ^∗∗∗^*p* < 0.001.

To verify the location of circNRIP1, we conducted the subcellular fractionation assay. The results demonstrated the main cytoplasmic distribution of circNRIP1 (Figure 2(a)). Afterwards, several potential binding microRNAs of circNRIP1 were predicted via TargetScan and miRanda. However, qRT-PCR depicted that only miR-653-5p level increased after knockdown of circNRIP1 in TPC-1 and K-1 cells (Figures 2(b) and 2(c)). Besides, miR-653-5p was depleted obviously in PTC tissues as well as PTC cell lines (Figures 2(d) and 2(e)). Thus, miR-653-5p was chosen for the present experiments. The binding fragment of miR-653-5p on circNRIP1 was obtained from StarBase and mutated accordingly (Figure 2(f)). Then, in order to determine the physical connection between circNRIP1 and miR-653-5p, we conducted luciferase reporter assay in TPC-1 and K-1 cells. The luciferase activity of circNRIP1-WT rather than circNRIP1-Mut decreased obviously under miR-653-5p mimics transfection (Figure 2(g)). Additionally, RNA pull down demonstrated miR-653-5p enrichment by biotinylated circNRIP1-WT compared with biotinylated circNRIP1-Mut (Figure 2(h)). To clarify the involvement of miR-653-5p in inhibitory effect of circNRIP1 knockdown on PTC, we successfully knocked down miR-653-5p through cotransfecting with miR-653-5p inhibitor (Figure 2(i)). Interestingly, EdU incorporation assay showed that the miR-653-5p depletion largely eliminated antiproliferation effect of si-circ-#1 (Figure 2(j)). Besides, the increased proportion of Tunel-positive cells induced by si-circ-#1 were counteracted through miR-653-5p inhibitor (Figure 2(k)). Moreover, transwell assay showed that miR-653-5p inhibition reversed the decrease in migration and invasion of PTC cells caused by silencing circNRIP1 (Figures 2(l) and 2(m)).

### 3.3. PBX3 Is a Direct Target of miR-653-5p

**Figure 3:**
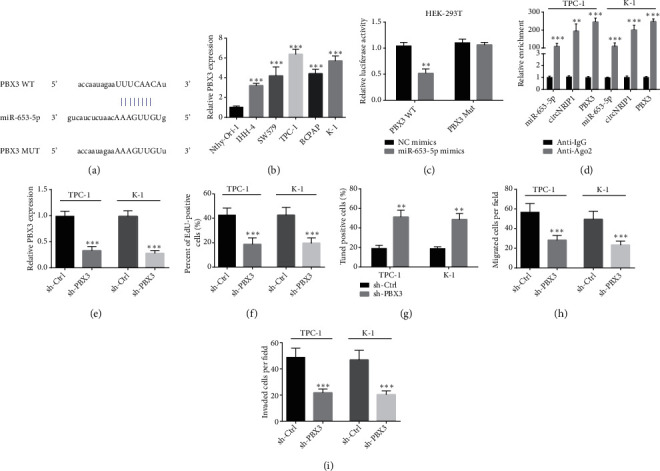
PBX3 is a direct target of miR-653-5p. (a) The binding site between miR-653-5p and the 3′-UTR of PBX3. (b) The PBX3 expression in PTC cell lines was determined by qRT-PCR assay. (c) Luciferase reporter assay was conducted to investigate the connection between miR-653-5p and PBX3 in HEK-293T. (d) RIP assay was carried out to determine the relationship among miR-653-5p, circNRIP1, and PBX3. (e) The success of PBX3 inhibition with sh-PBX3 in PTC cells was confirmed by qRT-PCR analysis. (f) EdU incorporation assay was used to detect the cell proliferation. (g) Tunel staining was used to evaluate the apoptosis of tumor cells. (h, i) The cell migration and invasion abilities were detected with transwell assay. ^∗^*p* < 0.05;  ^∗∗^*p* < 0.01;  ^∗∗∗^*p* < 0.001.

Subsequently, through bioinformatics analysis (TargetScan online tool), we explored the potential target of miR-653-5p, and found PBX3. The binding sequence of miR-653-5p and PBX3 3′-UTR is shown in Figure 3(a). Dramatically, PBX3 was elevated in PTC cell lines (Figure 3(b)). Luciferase reporter assay illustrated that luciferase activity was attenuated under cotransfection with miR-653-5p mimics and PBX3 WT (Figure 3(c)). We then performed RIP assay, and miR-653-5p, circNRIP1, and PBX3 presented enrichment by anti-Ago2, which further determined the relationship among miR-653-5p, circNRIP1 and PBX3 (Figure 3(d)). To study PBX3 role in PTC, we successfully inhibited PBX3 in PTC cells via transfecting with sh-PBX3 and furtherly verified by qRT-PCR assay (Figure 3(e)). EdU incorporation assay demonstrated that PBX3 inhibition significantly weakened PTC cell viability (Figure 3(f)). In addition, Tunel staining illustrated the increased PTC cell apoptosis ratio under PBX3 silencing (Figure 3(g)). Moreover, transwell assay revealed that PBX3 knockdown suppressed PTC cell migration and invasion (Figures 3(h) and 3(i)).

### 3.4. Overexpression of PBX3 Eliminates the Tumor Suppressive Effect of circNRIP1 Knockdown

**Figure 4:**
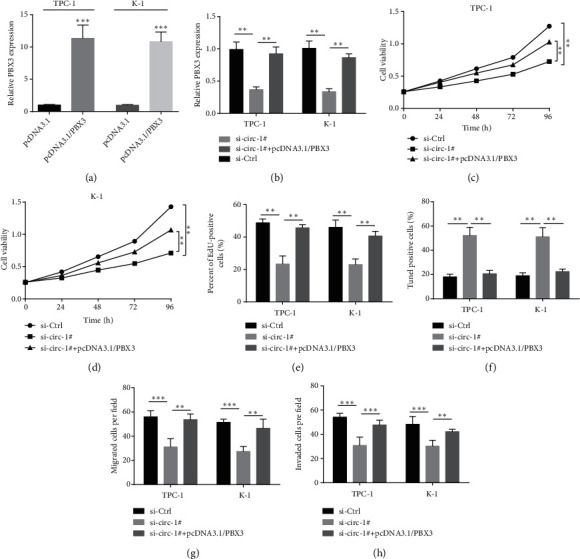
Overexpression of PBX3 eliminates the tumor suppressive effect of circNRIP1 knockdown. (a) The efficiency of pcDNA3.1/PBX3 was confirmed by qRT-PCR assay. (b) The PBX3 mRNA expression was evaluated by qRT-PCR assay. (c–e) CCK-8 and EdU incorporation assay were used to detect the cell proliferation. (f) Tunel staining was used to evaluate the apoptosis of tumor cells. (g, h) The cell migration and invasion abilities were detected with transwell assay. ^∗^*p* < 0.05;  ^∗∗^*p* < 0.01;  ^∗∗∗^*p* < 0.001.

To evaluate whether circNRIP1 promoted progression of PTC through miR-653-5p/PBX3 pathway, we furtherly conducted functional rescue experiments. qRT-PCR confirmed the efficiency of pcDNA3.1/PBX3 (Figure 4(a)) and revealed inhibitory effect of silencing circNRIP1 on PBX3 expression was recovered by pcDNA3.1/PBX3 transfection (Figure 4(b)). CCK-8 indicated that the decreased cell viability under circNRIP1 depletion was restored by PBX3 overexpression, which was consistent with the result of EdU incorporation assay exhibited (Figures 4(c)–4(e)). In addition, overexpression of PBX3 reversed the role of silencing of circNRIP1 on the apoptosis of tumor cells indicated by Tunel assay (Figure 4(f)). Moreover, the antimigration and anti-invasion roles of knockdown of circNRIP1 were abolished by pcDNA3.1/PBX3 transfection (Figures 4(g) and 4(h)).

### 3.5. circNRIP1 Knockdown Inhibits the Growth of PTC Tumor In Vivo

**Figure 5:**
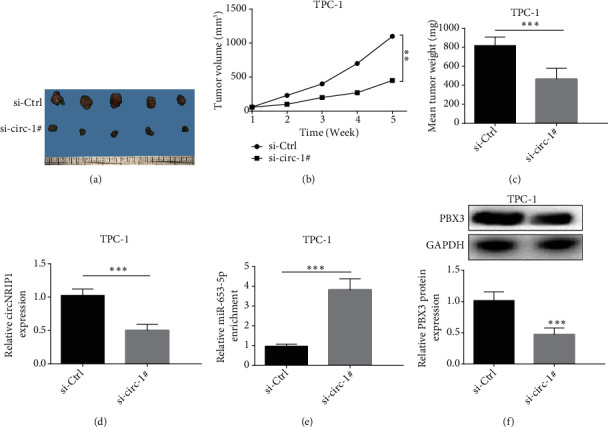
circNRIP1 knockdown inhibits the growth of PTC tumor in vivo. (a) The images of tumor in different groups. (b, c) The volume and the weight of tumors were recorded. (d, e) The expression levels of circNRIP1, miR-653-5p, and PBX3 protein were determined in tumor tissues. ^∗∗^*p* < 0.01;  ^∗∗∗^*p* < 0.001.

To clarify circNRIP1 role in PTC progression, we constructed xenograft models through injection with TPC-1 cells expressing si-Ctrl or si-circ-#1 into nude mice. The results demonstrated that tumor volume and weight decreased under circNRIP1 silencing (Figures 5(a)–5(c)). In addition, circNRIP1 and PBX3 expression decreased while miR-653-5p expression increased in tumor tissues under circNRIP1 knockdown (Figures 5(d) and 5(e)).

## 4. Discussion

Herein, circNRIP1 presented upregulation in PTC tissues and showed higher level in PTC with distant metastasis than in PTC without metastasis. The expression of circNRIP1 was also closely associated with TNM stage and overall survival rate of PTC. Moreover, we also showed that circNRIP1 level was significantly elevated in PTC cell line relative to in control cells. Knockdown of circNRIP1 suppressed proliferation, invasion, and migration of PTC cells and promoted apoptosis. Knockdown of circNRIP1 can significantly reduce the volume of PTC tumor *in vivo*. All these data indicate that circNRIP1 is an oncogene in PTC cells and exerts vital functions in PTC occurrence and development.

Tumorigenesis and progression result from many factors and go through a series of complex steps. Excessive proliferation and strong tumor cell invasion and migration are core initial steps of tumor development [25]. At present, some clinical targeted drugs mainly play roles in biological process of tumor cells, so as to weaken the progress of tumor [26]. Our study showed that knockdown of circRNA significantly suppressed proliferation/invasion/migration, whereas it promoted apoptosis of PTC cells. Consistent with our results, Xie et al. demonstrated that circ_0004771 knockdown attenuates breast cancer cell proliferation while promotes apoptosis [27]. Pan and Xu et al. observed overexpressed circ_0004771 in colorectal cancer and gastric cancer samples, and circ_0004771 may serve as a diagnostic biomarker [28, 29]. Ding and Huang et al. reported upregulated circ_0004771 in breast cancer and esophageal squamous cell cancer, and silencing of circ_0004771 could impede cell carcinogenic phenotypes and tumorigenesis [30, 31]. The above-mentioned result indicated that circNRIP1 was an oncogene, which can significantly affect the biological function of various tumor cells, including PTC.

One of the most extensive mechanisms of circRNA is ceRNA mechanism [32]. circRNA exert an effect on transcriptional regulation of downstream core genes through adsorbing and negatively regulating miRNAs [32, 33]. In this study, we obtained the target gene miR-653-5p of circNRIP1 based on biological information screening and RT-PCR analysis and proved that circNRIP1 can negatively regulate miR-653-5p. miR-653-5p depletion can significantly block the protective role of circNRIP1 silencing in PTC cell malignancy. miR-653-5p showed downregulation played a role in inhibiting the malignant phenotype of tumors, such as breast cancer, prostate cancer, cervical cancer, gastric cancer, lung cancer, ovarian cancer, and melanoma [34–40]. Consistent with a previous study [41], our study showed that miR-653-5p can also inhibit PTC cell proliferation and invasion, which further enriches our interpretation of miR-653-5p role in tumors.

Pre-B-cell leukemia homeobox 3 (PBX3) is the downstream of miRNA, which was verified by bioinformatics analysis and *in vitro* combination experiment [42]. PBX3, a dominant cofactor in the PBX family, can elevate DNA-binding/transcriptional activity of homeobox proteins [43]. In previous studies, Chen et al. have demonstrated that PBX3 showed elevation in PTC and PBX3 knockdown can inhibit PTC cell proliferation and invasion [42]. Herein, PBX3 overexpression successfully blocked the effects of circNRIP1 depletion on PTC cell malignancy, indicating that PBX3 mediates biological function of circRNAs in PTC.

In conclusion, circNRIP1 showed upregulation in PTC tissues and was related to lymphatic metastasis, tumor prognosis, and TMN stage. circNRIP1 inhibition promoted apoptosis whereas attenuated PTC cell proliferation/invasion/migration. Additionally, circNRIP1 silencing can reduce tumor size *in vivo.* Mechanistically, inhibition of circNRIP1 attenuated malignant progress of PTC through miR-653-5p/PBX3 axis, which may provide therapeutic target for PTC treatment.

## Figures and Tables

**Table 1:** Correlation between circNRIP1 expressions with clinical characteristics in PTC patients.

Characteristics	circNRIP1		*p* value
	Low	High	
Age (y)			0.821
≤60	21	26	
>60	10	11	
Gender			0.944
Male	19	23	
Female	12	14	
Lymph node status			0.008
Nonmetastasis	20	12	
Metastasis	11	25	
TNM stage			0.034
I-II	18	12	
III-IV	13	25	
Tumor size (cm)			0.001
≤1	21	10	
>1	10	27	

**Table 2:** Sequences of primers for PCR.

Genes	Forward primer	Reverse primer
circNRIP1	GGATCAGGTACTGCCGTTGAC	CTGGACCATTACTTTGACAGGTG
18S	CTTTCGTACGGTACGCCT	GCCAAGAGGGGCTTAGT
U6	TTGCTTCGTGTTTCTCAGT	AGTCCGGTTTTCCCCGAC
miR-330-5p	GTCTCTGGGCCTGTGTC	GTTGTGGTTGGTTGGTTTGT
miR-629-3p	GGGGTTCTCCCAACGTAAG	GAGGAGGAAGAAGAGGAGGA
miR-339-5p	TCCCTGTCCTCCAGGAG	GCGTTGTGTTGTGTTGTGTT
miR-653-5p	ACGCGTGTTGAAACAATCT	CCAACAACCAACCAACAACT
miR-595	CGAAGTGTGCCGTGGT	TCCTCCTCTCCTCTCCTCTC
PBX3	GACATCGGCGACATCCTCC	TCACACAGGACGCTGAAGAG

## Data Availability

The datasets analyzed during the current study are available from the corresponding author on reasonable request.
